# Letter from Dr. Ridley

**Published:** 1900-02

**Authors:** 


### CORRESPONDENCE.

*To Editors of the Atlanta Journal-Record of Medicine:*

From time to time there have appeared in THE ATLANTA JOURNAL-RECORD OF MEDICINE unwarranted attacks upon the State Board (Regular) of Medical Examiners. With the utmost courtesy to the editors of this periodical, for each of whom I have entertained personal regard, I feel that as president of this board I should reply to a recent article in its editorial.

I would call the attention of the editors to apparent errors.

The board does not yield precedence to the editors of THE ATLANTA JOURNAL-RECORD OF MEDICINE in the desire that the State of Georgia might rank above every other State in the personnel of its physicians; speaking professionally, we wish sincerely that Georgia was the "banner State." Personally, its board feels that it should receive its instructions relative to its duties, not from THE ATLANTA JOURNAL-RECORD OF MEDICINE, but rather from the "Act establishing boards of medical examiners for the State of Georgia, to define their duties, privileges, etc."

The legislature created the boards and defined clearly their duties, and the various boards find laid down for them in the act itself their whole official duty.

"The trouble is in our State Board of Medical Examiners."

This is a plain statement, yet it must have been prompted by ignorance or personal malice, for there cannot be found an expression in the act creating or in the oath binding the board which makes them the prosecutors in cases of illegal practice. As an official body, they examine applicants and issue licenses to such as pass satisfactory examinations, and when they do this their duty ceases and also their power. Personally, each member of the board stands on the same ground as any other reputable physician in this State, and I venture to say that each member has the honor of his profession as much in view as the editors of THE JOURNAL.

Officially, the board has no right to prosecute illegal practitioners. Its powers are not so defined.

The Act states in plain terms that the board is established for "the protection of the people from illegal and unqualified practitioners of medicine and surgery," and not as the editor of THE ATLANTA JOURNAL-RECORD OF MEDICINE claims, "and yet Georgia has a law to protect the medical profession and put all comers on the same footing."

The remarks which the editor makes so recklessly might justly be construed as the result of personal pique, or simply a case where he feels he must say something, and selects a subject which, it is to be hoped at least, he knows least about. Now as to the real trouble.

The Board of Examiners officially has no duty except to pass upon the competency of applicants and issue licenses. Each citizen of Georgia is morally obligated to report and aid in the suppression of any breach of law. A breach of law relative to the practice of medicine is a misdemeanor, and it is the duty of each citizen who may know of such illegal practice to report it to the grand jury and have the person so practicing indicted. After that is done it becomes the duty of the solicitors of Georgia to prosecute the person so charged. An infringement of this law, prohibiting the illegal practice of medicine does not in any respect differ from the breaking of any other law. It is simply a crime against the State, and it is the privilege and moral duty of any citizen knowing of such crime to present the criminal to the proper authorities.

The main trouble that has prompted THE ATLANTA JOURNAL-RECORD OF MEDICINE, it seems, is that Atlanta suffers most. I respectfully submit that it is the province of the editors of THE ATLANTA JOURNAL-RECORD OF MEDICINE, and other physicians and citizens of Atlanta, to report these violations of law and have the guilty punished, and if the physicians resident who are so prompt to criticize have not the courage to discharge their duty as members of the profession and as citizens of the State, then they ought not to expect the Board of Examiners to stand in front of them. If they know the facts it is their duty to relate them to the grand jury; if they do not know them, then the editor, who is responsible, should be just and generous enough to correct his criticism of the board.

In conclusion I would say that the law under discussion is not perfect, and one of its notable deficiencies is, that it does not provide a punishment for those who would criticize without knowledge or investigation.

F. M. RIDLEY, M.D.,
President State Board Medical Examiners.

LaGrange, Ga., December 28, 1899.

---

NEW YORK, January 22, 1900.

*Atlanta Journal-Record of Medicine,*
*Atlanta, Ga.*

GENTLEMEN:—Our attention has been called to a misprint in the third edition of The Newer Remedies, by Professor Virgil Coblentz, whereby the dose of Blennostasine is given as from 1 to 4 grammes (15 to 60 grains) instead of from 1 to 4 grains.

We have called the author's attention to the misprint, and in the reprint of the third edition an erratum has been inserted correcting the dose. Inasmuch as the work has been widely circulated prior to correction, we would ask you through the medium of your valued journal to call the attention of the profession to the error, as the administration of so large a dose would, in all probability, cause serious complications. Very respectfully yours,

MCKESSON & ROBBINS.

P. S.—We would also state that we have a number of copies of The Newer Remedies for distribution to the profession, and will be pleased to send a copy to any of your readers on receipt of card.

IT would seem that in the present war the Boer hospital service, concerning which much has been said in depreciation, is in reality very well equipped. The Transvaal has the orthodox Red Cross Society and an efficient St. John's Ambulance Society as auxiliaries to the regular military medical corps. The latter is well equipped, although numerically weak. Several railroad trains have been made ready, fitted with swinging beds and all modern conveniences for alleviating suffering. The women of Pretoria and Johannesburg responded nobly to the invitation to do volunteer duty in nursing "for the sake of their Lord and their country," as the pathetic appeal reads. It is said the English nurses have been put out of the hospitals at Johannesburg, the physician in charge insisting that "the English women are not fit to nurse the Dutch."—*Medical News.*

